# Prognostic value of estrogen receptor-α and progesterone receptor in curatively resected colorectal cancer: a retrospective analysis with independent validations

**DOI:** 10.1186/s12885-019-5918-4

**Published:** 2019-10-07

**Authors:** Shu-Biao Ye, Yi-Kan Cheng, Lin Zhang, Xue-Ping Wang, Lei Wang, Ping Lan

**Affiliations:** 1 Department of Colorectal Surgery, The Sixth Affiliated Hospital, Sun Yat-sen University, Guangzhou, Guangdong People’s Republic of China; 2 Guangdong Provincial Key Laboratory of Colorectal and Pelvic Floor Diseases, the Sixth Affiliated Hospital, Sun Yat-sen University, Guangzhou, Guangdong People’s Republic of China; 3 Department of Radiation Oncology, The Sixth Affiliated Hospital, Sun Yat-sen University, Guangzhou, 510655 People’s Republic of China; 4 Department of Clinical Laboratory, Sun Yat-sen University Cancer Center, Guangzhou, 510060 People’s Republic of China; 5 State Key Laboratory of Oncology in South China, Guangzhou, 510060 People’s Republic of China; 6 Collaborative Innovation Center for Cancer Medicine, Guangzhou, 510060 People’s Republic of China

**Keywords:** Colorectal cancer, Curative resection, Prognosis, Estrogen receptor-α, Progesterone receptor

## Abstract

**Background:**

Prognostic assessment is crucial for optimal treatment. The aim of our study was to investigate the potential impact of estrogen receptor-α (ER-α) and progesterone receptor (PR) on the prognosis of colorectal cancer (CRC) patients who received curative resection.

**Methods:**

Retrospective evaluation of two independent cohorts of CRC patients maintained prospectively in 2009–2010 (training set) (*n* = 148) and 2007–2009 (internal validation set) (*n* = 485). Furthermore, we used an external independent CRC cohort from The Cancer Genome Atlas (TCGA) (*n* = 511) for further validation. ER-α and PR expression as well as other potential prognostic factors were retrospectively evaluated in training set with respect to overall survival (OS), local relapse free survival (LRFS) and distant metastasis free survival (DMFS). The prognostic factors found in training set will be validated in two validation cohorts.

**Results:**

On univariate analysis for the training set, OS, LRFS and DMFS were not associated with PR expression. While patients with ER-αexpression were found to have poor prognosis. In addition, multivariate analysis showed that ER-αexpression maintained significance with respect to OS (HR, 5.06; *p* = 0.002), LRFS (HR, 8.81; *p* = 0.002) and DMFS (HR, 8.07; *p* = 0.004). Similarly, ER-α expression showed prognostic significance with respect to OS with hazard ratios (HRs) of 1.572 (95% CI: 1.001–2.467, *p* = 0.049) and 1.624 (95% CI: 1.047–2.520, *p* = 0.031) for the internal and external validation cohort, respectively.

**Conclusion:**

ER-α expression was a biomarker of poor prognosis and it might inform treatment decision for high risk CRC patients. However, PR expression was not associated with survival outcomes.

**Electronic supplementary material:**

The online version of this article (10.1186/s12885-019-5918-4) contains supplementary material, which is available to authorized users.

## Background

Colorectal cancer (CRC) is the third most common cancer and ranks the third in the causes of cancer mortality in the world [1]. Owing to the change of lifestyle, the incidence and mortality of CRC are rising rapidly in developing countries [1]. The survival outcome of CRC is not promising mainly due to local recurrence and distant metastasis [2]. Tumor markers are potentially useful in prediction of prognosis and formation of treatment strategy [3]. The TNM staging system provides a useful benchmark for aiding diagnosis, determining prognosis and monitoring treatment. However, patients with same stage and similar treatment regimen may have different clinical outcomes. Considering these, it is crucial to investigate new prognostic biomarkers to reflect the biological heterogeneity of cancer and then to identify high risk CRC patients.

Recent investigations have suggested that the tumor cell expression of hormone receptors may have an impact on the prognosis of patients with CRC [4]. A study from Germany indicated that lack of estrogen receptor (ER)-β was independently associated with poor survival [5]. Although ER-α was reported to be implicated in the development and progression of colorectal cancers according to Caiazza et al. [6] and Nussler et al. [7], the prognostic value of ER-α in CRC needs to be investigated. Furthermore, a study from the United States suggested that the expression of PR by the tumor cells may be associated with a shorter patient survival [8].

Epidemiologic investigations indicated that men are more likely to develop CRC at all stage than women [9, 10]. In addition, a meta-analysis conducted by Harvard University [11] and a population-based case-control study from German [12] demonstrated that exposure to exogenous hormones has been found to be associated with a reduced risk for CRC in postmenopausal women. Thus, ER and PR may play a role in the genesis and development of CRC; but the prognostic value of ER-α and PR expression in CRC patients remains unclear.

It is increasingly recognized that variations within clinical outcome in cancer patients are affected by not only the oncological characteristics such as stage but also the host factors. Thus, investigating the potential prognostic impact of tumor cell expression of hormone receptors combining with other host clinical characteristics appears important. The aim of this study was therefore to investigate the potential prognostic impact of tumor cell expression of hormone receptors in terms of local relapse-free survival (LRFS), distant metastasis-free survival (DMFS), and overall survival (OS) in patients who received curative resection for non-metastatic CRC and further to validate the prognostic role in two independent CRC datasets.

## Methods

### Study design and patients

#### Training set

Subjects were included from November 2009 until October 2010. Those eligible for.

inclusion were patients who underwent curative resection for non-metastatic CRC at Sun Yat-Sen University Cancer Center. Tumor tissue from 148 patients was available and the prognostic value of ER-α and PR were determined by immunohistochemistry.

#### Internal validation set

Internal validation set consisted of patients referred consecutively with a confirmed diagnosis of non-metastatic CRC. This cohort of 485 patients was included at registries from September 2007 October 2009.

For training and internal validation cohorts, patient data regarding: demography, tumor stage, tumor localization and survival were retrieved from patient files and registries.

#### External validation set

Findings from the above both cohorts were compared to a publicly available, open-access, dataset of CRC from The Cancer Genome Atlas (TCGA) (https://tcga-data.nci.nih.gov/tcga/) (March 16th, 2017 update). For external validation set, patient data regarding: ER-α expression level, demography, tumor stage, tumor localization and survival were available.

### Immunohistochemistry

The tissue samples from training and internal validation cohorts were obtained from resected specimens. The tissues were fixed in 10% buffered formalin (PH7.0) and embedded in paraffin. The paraffin-embedded tumor samples were sectioned continuously into 4-μm-thick sections. Then the sections were dewaxed in xylene and rehydrated in graded alcohols. A negative control was performed by replacing the primary antibody with a normal rabbit IgG antibody. Following antigen retrieval by microwave heating (95 °C for 20 min), sections were then incubated with primary monoclonal rabbit anti-human PR (Clone 1E2, Ventana Medical Systems. Inc) (for training set only) or ER (Clone SP1, Ventana Medical Systems. Inc) at 4 °C overnight. After washing, the sections were incubated with a horseradish peroxidase-labeled goat antibody against a mouse/rabbit secondary antibody (Envision; Dako, Glostrup, Denmark) at room temperature for 30 min. Then the signal was developed with 3, 3′-diaminobenzidine tetrahydrochloride (DAB). Section was counterstained with hematoxylin. The stain was examined by two pathologists independently. The data were obtained by calculating the mean number of positively stained cells in five to ten separate 400 × per high power field(HPF) and evaluated as negative or positive. Two independent observers blinded to the clinicopathological information scored the ER-α and PR expression levels in tumor cells by assessing (a) the proportion of positively stained cells: (0, < 5%; 1, 6 to 25%; 2, 26 to 50%; 3, 51 to 75%; 4, > 75%) and (b) the signal intensity: (0, no signal; 1, weak; 2, moderate; 3, strong). The score was the product of a × b. Considering that the number of patients with score > 0 in ER-α and PR expression were 19 (12.8%) and 32 (21.6%), we used dichotomic classification (positive/negative). Therefore, the patients were divided into subgroups: a high group (a × b > 0) and a low group (a × b = 0). The score a and b were the averages of scores of two independent observers. Immunohistochemical staining was showed in Additional file 1: Figure S1.

### Treatment

For training and internal validation cohorts, the final decisions with regard to treatment strategy and use of chemotherapy or radiotherapy were based on TNM stage, the multidisciplinary team’s (MDT) decision and patient choice. All patients were treated with definitive-intent surgery. Most of the patients with stage II-III rectal cancer received radiotherapy. 5-FU based chemotherapy was delivered concurrently with radiation in forms of three-dimensional conformal radiotherapy (3D-CRT), intensity-modulated radiotherapy (IMRT) or Volumetric Modulated Arc Therapy (VMAT). Neoadjuvant or adjuvant chemotherapy consisted of oxaliplatin (130 mg/m^2^, day 1) with capecitabine (1000 mg/m^2^, bid, days 1–14) every 3 weeks to a total of 6 months’ perioperative therapy or Leucovorin (400 mg/m^2^ or 200 mg/m^2^, day1), with 5-fluorouracil (5-FU) (bolus 400 mg/m^2^ and then 1200 mg/m^2^/day over 46-48 h) every 2 weeks to a total of 6 months’ perioperative therapy. Of 148 patients, 12 (8.1%) patients received neoadjuvant chemotherapy and 86 (58.1%) patients received adjuvant chemotherapy. All patients from external validation cohort (TCGA dataset) underwent surgery.

### Follow-up

Follow-up for training and internal validation cohorts was measured from the first day of treatment until the day of last examination or the day of death. Patients were evaluated every 3 months for the first 2 years, and then every 6 months for the next 3 years and finally annually. Relapse local disease was diagnosed pathologically by surgical resection, biopsy or cytology and/or by the detection of radiologically obvious lesions that increased in size over time. Additional tests were ordered when indicated to identify distant failure.

### Statistical analysis

The cut-off values in external validation cohort (TCGA cohort) were obtained using X-tile (Version 3.6.1, Yale University, New Haven, CT). All survival analyses were done with STATA 12 statistical software (Stata Corp LP, College Station, Texas, USA). OS, DMFS and LRFS were all estimated by the Kaplan-Meier method and the survival curves compared using the log-rank tests. The following outcomes of interest (interval to the first defining event) were evaluated: OS, LRFS and DMFS. We calculated OS from commencement of treatment to death or the date of last follow-up visit for surviving patients. The latencies (i.e.*,* time from commencement of treatment) to the first local or remote relapse were calculated for LRFS and DMFS, respectively.

Multivariate analyses using the Cox proportional hazards model were used to estimate the hazard ratios (HR) and test independent significance by backward elimination of insignificant explanatory variables. Covariates included host factors (i.e., sex, age,), and tumor factors (i.e., tumor localization, stage), the criterion for statistical significance was set at *p* = 0.05 and *p* values were based on 2-sided tests.

## Results

### Patient characteristics

The median follow-up duration was 46.8 months (3.1–73.5 months) for training cohort, 64.7 months (0.2–150.1 months) for internal validation cohort and 23.8 months (0.2–105.1 months) for external validation cohort, respectively. The patients’ baseline characteristics of three cohorts are presented in Table 1.

**Table 1:** Baseline characteristics

	Training set	Internal validation set	External validation set
Number of patients	148	485	511
Patient demographics			
Median age (min-max)	58 (16–89)	59 (16–86)	68 (31–90)
Male (number, %)	91 (61.5%)	290 (59.8%)	270 (52.8%)
Localization			
Colon	77 (52.0%)	221(45.6%)	379 (74.2%)
Rectum	71 (48.0%)	264 (54.4%)	132 (25.8%)
Stage			
Stage I	22 (14.9%)	99 (20.4%)	106 (20.7%)
Stage II	64 (43.2%)	178 (36.7%)	224 (43.8%)
Stage III	62 (41.9%)	208 (42.9%)	181 (35.4%)
ER-α expression			
High (number, %)	19 (12.8%)	78 (16.1%)	158 (30.9%)
Low (number, %)	129 (87.2%)	407 (83.9%)	353 (69.1%)
PR expression			
High (number, %)	32 (21.6%)		
Low (number, %)	116 (78.4%)		

Baseline characteristics of the two cohorts regarding demography (age and gender), tumor features (stage and localization), ER-α expression and PR expression (just in training set)

*Abbreviations*: *ER-α* estrogen receptor –α, *PR* progesterone receptor

### Impact of tumor expression of ER-α and PR on survival outcomes in training cohort

**Fig. 1:**
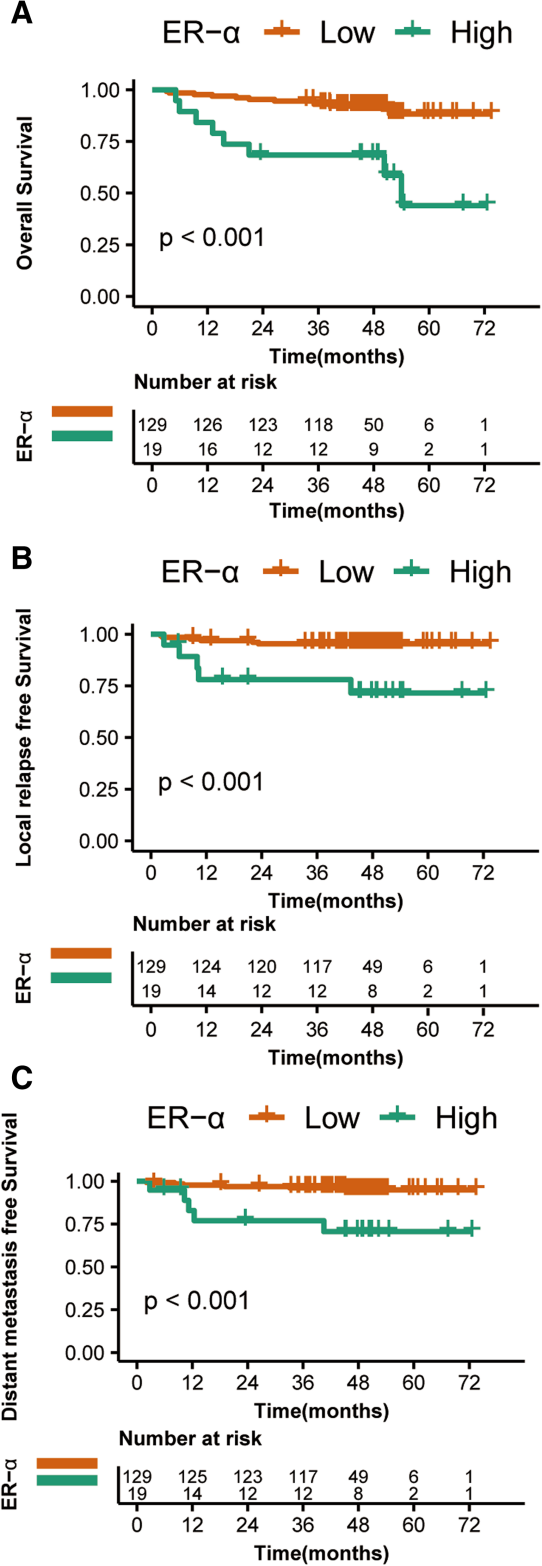
Kaplan-Meier survival curves for the patients with CRC patients from training cohort in the high ER-α expression group and low expression group. **a** Overall survival, **b** distant metastasis-free survival, **c** local relapse-free survival

To investigate the effect of tumor expression of ER-α, PR on the outcomes of patients with CRC, the 5-year actuarial OS, DMFS and LRFS rates in training cohort were analyzed. On univariate analysis, low and high ER-α expression demonstrated significant differences in the 5-year OS (89% vs. 47%, *p* < 0.001), LRFS (95% vs. 71%, *p* < 0.001) and DMFS (95% vs. 70%, *p* < 0.001) (Fig. 1, Tables 2 and 3) rates of CRC patients, while PR were not found to be significantly associated with improved 5-year OS, LRFS and DMFS rates (Additional file 1: Table S1).

**Table 2:** Overall survival analyses for training and validation cohorts

	Training set		Internal validation set		External validation set	
	Hazard ratio (95% CI)	*P* value	Hazard ratio (95% CI)	*P* value	Hazard ratio (95% CI)	*P* value
Univariate						
ER-α						
High vs. low	5.221 (2.073–13.146)	< 0.001*	1.596 (1.020–2.497)	0.039*	1.774 (1.145–2.749)	0.02*
PR						
High vs. low		0.869				
Multivariate						
Age						
> 60y vs. ≤60y	0.462 (0.178–1.196)	0.112	1.111 (0.762–1.621)	0.584	2.029 (1.149–3.585)	0.015*
Gender						
Female vs. male	1.143 (0.427–3.062)	0.790	0.890 (0.605–1.309)	0.554	1.154 (0.747–1.781)	0.519
Stage						
Stage II- III vs. I	2.841 (1.230–6.558)	0.014*	1.715 (1.287–2.287)	0.000*	2.654 (1.153–6.110)	0.022*
Localization						
Rectum vs. colon	0.909 (0.354–2.333)	0.842	1.861 (1.271–2.727)	0.001*	1.140 (0.667–1.947)	0.632
ER-α						
High vs. low	5.061 (1.833–13.968)	0.002*	1.572 (1.001–2.467)	0.049*	1.624 (1.047–2.520)	0.031*

Uni- and multivariate survival analyses for risk of death. Hazard ratios were calculated by the adjusted Cox proportional hazards model

*Abbreviations*: *ER-α* estrogen receptor –α, *PR* progesterone receptor

*statistically significant

**Table 3:** Local recurrence-free survival and distant metastasis-free survival analyses for training and internal validation cohorts

	LRFS (HR, 95%CI, *p* value)		DMFS (HR, 95%CI, *p* value)	
	Training set	Internal validation set	Training set	Internal validation set
Univariate analysis				
ER-α				
High vs. low	6.63 (2.02–21.77), < 0.001*	1.85 (0.73–4.69), 0.174	6.79 (2.07–22.29), < 0.001*	1.346 (0.822-2.203), 0.329
Multivariate analysis				
Age				
> 60y vs. ≤60y	0.40 (0.11–1.49), 0.170	0.70 (0.30–1.62), 0.444	0.28 (0.07–1.09), 0.067	0.94 (0.63–1.40), 0.914
Gender				
Female vs. male	0.63 (0.16–2.47), 0.503	0.94 (0.41–2.18), 0.892	1.56 (0.43–5.70), 0.497	1.20 (0.80–1.79), 0.373
Stage				
Stage II- III vs. I	3.61 (1.12–11.65), 0.032*	1.09 (0.64–1.87), 0.196	3.56 (1.11–11.39), 0.032*	1.33 (1.02–1.73), < 0.001
Localization				
Rectum vs. colon	0.94 (0.26–3.44), 0.926	0.30 (0.10–0.89), 0.028	0.69 (0.20–2.35), 0.552	1.50 (0.92–2.24), 0.076
ER-α				
High vs. low	8.66 (2.24–33.41), 0.002*	1.71 (0.67–4.35), 0.285	6.61 (1.85–23.60), 0.004*	1.30 (0.79–2.13), 0.451

Uni- and multivariate survival analyses. Hazard ratios were calculated by the adjusted Cox proportional hazards model

*Abbreviations*: *ER-α* estrogen receptor -α

*statistically significant

In the COX multivariate analysis, the following parameters were included: age (< 60 years,≥60 years), sex, location of tumor, stage (I, II-III) tumor expression of ER-α. Stage maintained statistical significance in OS. In addition, ER-α expression was an independent prognostic factor for OS in CRC patients with surgery (HR,5.061; *p* = 0.002) (Table 2), LRFS (HR, 8. 655; *p* = 0.002) and DMFS (HR, 6.610; *p* = 0.004) (Table 3).

### Validation of prognostic value of ER-α expression on survival outcomes in internal and external validation cohorts

**Fig. 2:**
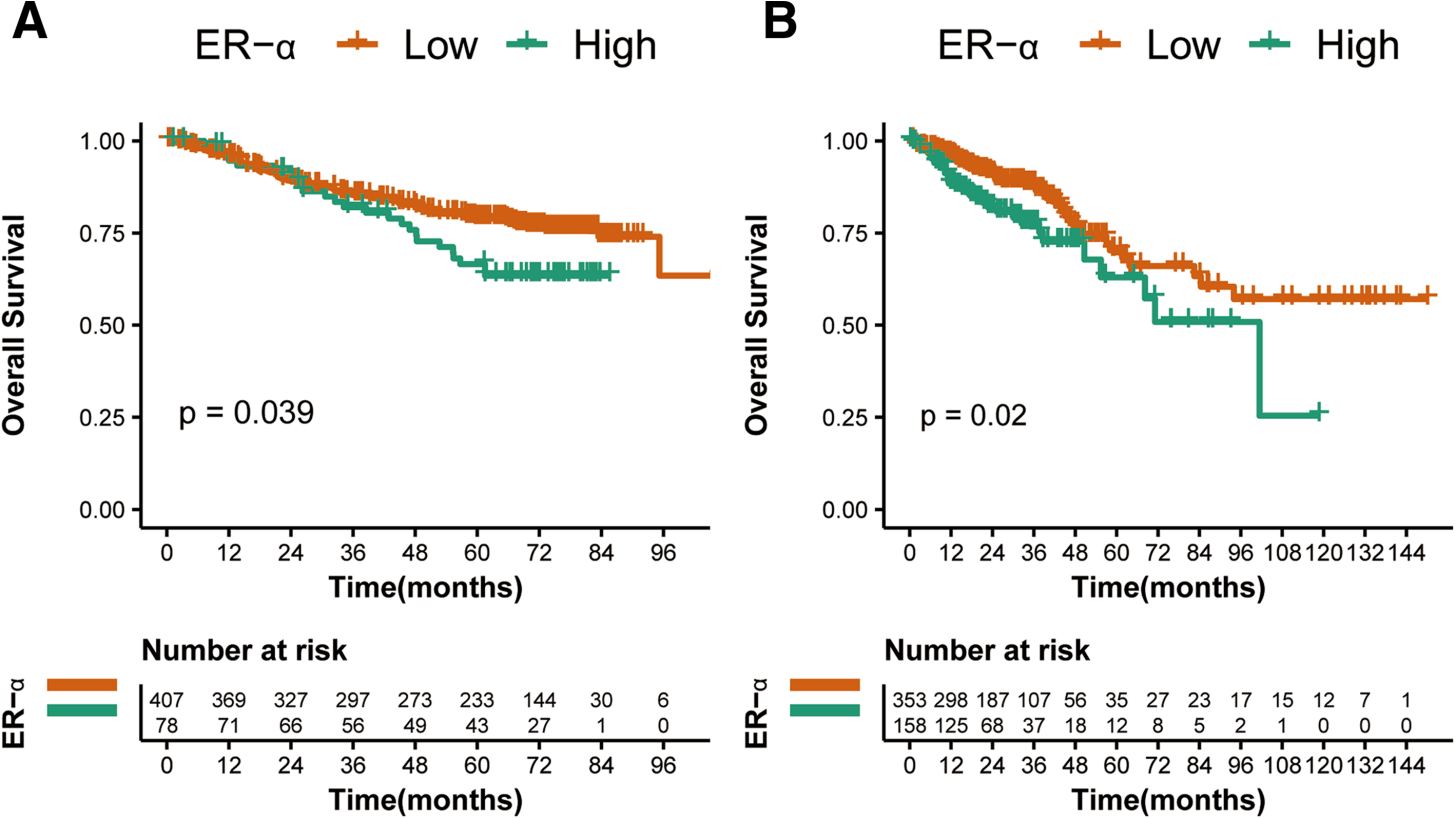
Kaplan-Meier survival curves of overall survival for the patients with CRC patients. **a** Internal validation cohort for high ER-α expression group and low expression group, **b** External validation cohort for high ER-α expression group and low expression group

To validate the prognostic value of ER-α, the 5-year actuarial OS, DMFS and LRFS rates in internal validation cohort and the 5-year actuarial OS rates in external validation cohort were analyzed. On univariate analysis, tumor expression of ER-α demonstrated significant differences in the 5-year OS rates in internal and external validation cohorts, which are 74% vs. 61% with *p* = 0.039 and 53% vs. 38% with *p* = 0.02 (Fig. 2), respectively. Whereas, univariate analyses indicated that ER- α expression had no significant association with DMFS and LRFS in internal validation cohort (Table 3). Since there is no data about local recurrence and distant metastasis in external validation cohort (TCGA dataset), DMFS and LRFS were not validated in this set.

In the COX multivariate analysis, ER-α expression was an independent prognostic factor for OS in both validation cohorts, with HR = 1.572, 95%CI (1.001–2.467), *p* = 0.049 and HR = 1.624, 95%CI (1.047–2.520), *p* = 0.031 for internal and external validation sets (Table 2).

## Discussion

Prognostic assessment is crucial for optimal treatment. In routine clinical practice, the TNM staging system is the most important prognostic determinant for the treatment strategy in CRC patients. However, patients with the same stage have been reported to have various survival outcomes, which suggests that identifying more potential prognostic markers are necessary. We investigated the prognostic value of tumor cell expression of ER-α and PR in CRC patient. And the results demonstrated that ER-α expression was predictive of survival of CRC patients independent of stage, allowing clinicians to potentially identify high risk patients for more intensive treatment to improve survival outcomes. More importantly, the prognostic value of ER-α expression was confirmed by independent internal and external CRC datasets in our study in spite of differences in expression due to distinct genetic background and analytic methods. However, the results in training cohort did not indicate the clinical validity of PR expression as a prognostic biomarker.

ER-α can be used as prognostic biomarker in many types of cancer and might be implicated to tumor progression of CRC [13]. Therefore, we aim to investigate the potential impact on prognosis in patients with CRC. In gastric cancer, ER-α expression is generally an indicator for a poor prognosis [14] which we anticipated would be the same case in CRC. Our study found that ER-α expression was a negative prognostic factor as it was in lung cancer and hepatocellular carcinoma [15, 16]. These studies implied that ER-α mediated antiapoptotic signal ways might be one of reasons for poor survival [17]. Otherwise, loss of ER-β in CRC has been associated with advanced cancer stages and poor prognosis [5, 18, 19]. In addition, decreased ER-β expression concurrent with increased ER-α expression have been reported to play a key role on cancer development and advanced stages [13, 20]. Therefore, the prognostic impact of ER-α and ER-β appears to be different in CRC, which occurs as well in other gastrointestinal tumors like gastric cancer [14] and esophageal cancer [21]. Our findings suggested that PR expression was not a prognostic factor in CRC patients. According to Heijmans et al., PR signaling has no role in intestinal tumorigenesis, which indicated that PR expression may contribute little to tumor genesis and development [22].

At present, the standard treatment for locoregionally advanced CRC is surgery with neoadjuvant or adjuvant chemoradiotherapy, and local or distant relapses occurred in almost 50% of patients. This pattern of failure suggests that certain subgroup of patients do not benefit from present strategies. Thus, the accurate identification of subgroups of patients lead to more individualized therapy. Patients with ER-α expression had poorer survival than those without ER-α expression, and therefore further studies are needed to identify more intensive systemic approaches to improve the treatment outcomes of patients with ER-α expression.

The present study with validation from two independent CRC datasets indicated that ER-α expression was a prognostic factor independent of stage, leading to personalized therapy. When interpreting the results, the retrospective nature of the present study and the heterogeneity of three cohorts should be considered. Hence, we acknowledge that prospective, large-scale, multicenter studies are necessary to confirm our results. In addition, the mechanism behind the prognostic value in CRC is unclear. Further studies on the role of ER-α in CRC are warranted.

## Conclusion

ER-α expression was a marker of poor prognosis and it might inform treatment decision for high risk CRC patients. However, PR expression was not associated with survival outcomes.

## Additional file

Additional file 1: **Figure S1.** Immunohistochemical staining for estrogen receptor-α (ER-α) and progesterone receptor (PR) in human colorectal cancer. Our data showed high expression of ER-α (A, X 100; B, X 400) and high expression of PR (C, X 100; D, X 400) low expression (E, X 100; F, X 400) in tumor tissues from patients with CRC. **Table S1.** Univariate survival analysis of ER-α and PR expression in training cohort. (ZIP 8731 kb)

## Data Availability

All data generated and/or analyzed during this study are available from the corresponding author upon reasonable request.

## Abbreviations

3D-CRT: Three-dimensional conformal radiotherapy
CRC: Colorectal cancer
DMFS: Distant metastasis-free survival
ER-α: Estrogen receptor-α
HR: Hazard ratios
IMRT: Intensity-modulated radiotherapy
LRFS: Local relapse-free survival
OS: Overall survival
PR: Progesterone receptor
TCGA: The Cancer Genome Atlas
VMAT: Volumetric Modulated Arc Therapy
