# Supplementary figures and images for: Prognostic value of estrogen receptor-α and progesterone receptor in curatively resected colorectal cancer: a retrospective analysis with independent validations
Source: BMC Cancer. 2019 Oct 7;19:933. doi: 10.1186/s12885-019-5918-4 (PMC6781392; doi:10.1186/s12885-019-5918-4)

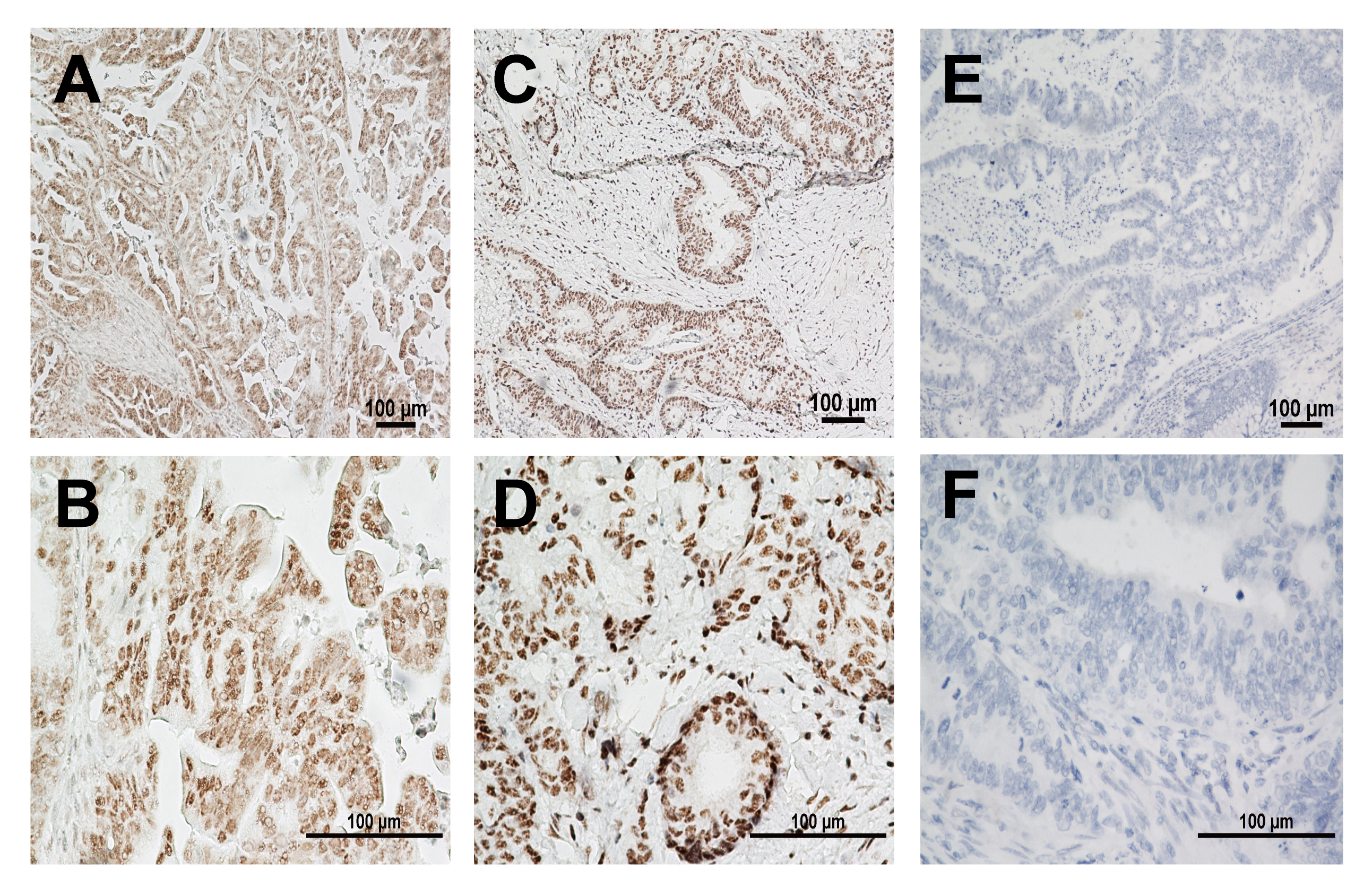

Supplement: Supplementary file 1 — Figure S1. Immunohistochemical staining for estrogen receptor-α (ER-α) and progesterone receptor (PR) in human colorectal cancer. Our data showed high expression of ER-α (A, X 100; B, X 400) and high expression of PR (C, X 100; D, X 400) low expression (E, X 100; F, X 400) in tumor tissues from patients with CRC. Table S1. Univariate survival analysis of ER-α and PR expression in training cohort. (ZIP 8731 kb) [file 12885_2019_5918_MOESM1_ESM.zip › S Figure 1R4.tif]
