# Supplementary material for: Prognostic value of estrogen receptor-α and progesterone receptor in curatively resected colorectal cancer: a retrospective analysis with independent validations
Source: BMC Cancer. 2019 Oct 7;19:933. doi: 10.1186/s12885-019-5918-4 (PMC6781392; doi:10.1186/s12885-019-5918-4)
Supplement: Supplementary file 1 — Figure S1. Immunohistochemical staining for estrogen receptor-α (ER-α) and progesterone receptor (PR) in human colorectal cancer. Our data showed high expression of ER-α (A, X 100; B, X 400) and high expression of PR (C, X 100; D, X 400) low expression (E, X 100; F, X 400) in tumor tissues from patients with CRC. Table S1. Univariate survival analysis of ER-α and PR expression in training cohort. (ZIP 8731 kb) [file 12885_2019_5918_MOESM1_ESM.zip › revised supplementary materialR4.doc]

Additional file 1

**Table S1 Univariate survival analysis of ER-α and PR expression in training cohort**

|  | OS at  5 years, % | Hazard Ratio†  (95% CI*) | p‡ | LRFS at  5 years, % | Hazard Ratio (95% CI) | P | DMFS at  5 years, % | Hazard Ratio^a†^  (95% CI) | | p |
| --- | --- | --- | --- | --- | --- | --- | --- | --- | --- | --- |
| ER-α expression |  | 1.85  (1.24-2.78) | 0.002 |  | 2.78  (1.36-5.71) | 0.004 |  | 1.65  (1.05-2.60) | 0.029 | |
| Low | 89 |  |  | 95 |  |  | 95 |  |  | |
| High | 47 |  |  | 71 |  |  | 70 |  |  | |
| PR expression |  | 1.04  (0.68-1.59) | 0.869 |  | 0.69  (0.27-1.78) | 0.437 |  | 0.85  (0.59-1.40) | 0.526 | |
| Low | 76 |  |  | 93 |  |  | 78 |  |  | |
| High | 63 |  |  | 95 |  |  | 79 |  |  | |

*CI, confidential internal

† Hazard ratios were calculated by the adjusted Cox proportional hazards model.

‡ p was calculated by the unadjusted log-rank test.

Abbreviations: ER=estrogen receptor; PR=progesterone receptor; OS=overall survival; LRFS=local relapse free survival; DMFS=distant metastasis free survival; HR=hazard ratio; CI=confidence interval; NS=not significant.

**Figure S1.** Immunohistochemical staining for estrogen receptor-α (ER-α) and progesterone receptor (PR) in human colorectal cancer. Our data showed high expression of ER-α (A, X 100; B, X 400) and high expression of PR (C, X 100; D, X 400) low expression (E, X 100; F, X 400) in tumor tissues from patients with CRC.
